# A Brief Epistle to the Esculapians

**Published:** 1908-05

**Authors:** 


### EDITORIAL DEPARTMENT.

### A Brief Epistle to the Esculapians.

I take it that doctors get tired of a steady diet of "interesting cases," of "society reports" and of fever, pus and bile; and that, like our horses fed all winter on dry forage, they want a change of diet. Especially in the spring time do both, man and cattle, relish a little "greenness"—grass and garden truck. The "Red Back" for many years has catered to this appetite, and while it has not given its readers any "green stuff," I hope, it has given them strong, vigorous papers by the ablest men, and rare spiced dishes editorially and other "relishes,"—a change. The present issue will be found especially spicy. Dr. Lydston's robust socio-sanito-political paper and "Mrs. Pluto's" letter to Dr. Worsham will be found refreshing.

And, by the bye, the "Red Back," for nearly twenty-five years, has been the friend, ally, advocate and defender of the regular profession, the uncompromising foe to quackery and quacks, the champion of organized medicine. It was one of the leading and principal factors in the reorganization and upbuilding of our splendid society. It was all this before the State Association journal, now a gratifying success and a credit to the profession and the State, was born. From the nature of the organization every member is a subscriber to the *State Journal, nolens volens,* half of his dues to the Association going to pay his subscription. But that is no reason why every member should not, also, support the "Red Back." It is being boycotted by some, with whom we differ on certain matters, and the late ruling of the Postoffice Department is especially hard on it, because, appreciating the fact that in many portions of Texas everything is done on long credit and doctors collect, as a rule, but once a year, it has extended unlimited credit to hundreds of its subscribers—pushing none for payment. The Department orders that delinquent subscribers who do not renew their subscriptions within four months from notice will not be counted as subscribers and the mails will be closed to all such. I appeal to those who owe me to take advantage of the Corpus Christi meeting, or other early opportunity, to stand by their old "stand-by," and to ante and get a new deal, if they would not see the old favorite and faithful friend go to the wall. And I appeal to those who have not enjoyed its strong, independent, original and racy pages in the past—the younger set—to catch on now. The "Red Back" is *sui generis*. It is unlike any other journal. It does not feed you forever on interesting cases, fever, pus and bile, but throws into every issue a little "nutmeg, cinnamon, spice and cloves" (and that's what gives it its jolly red nose), as *Mater Anser* wisely remarks.

Seriously, there is room for and need of an independent medical journal in Texas; one unhampered and free to give both sides, and the "Red Back" feels that it has earned the continued support of its old friends,—many of twenty-three years standing,—as well as of all who believe in a square deal. Subscribe for the "Red Back." Renew your subscription, if you would not see your old friend crushed out.

